# Syphilis in Different Countries

**Published:** 1881-12

**Authors:** 


### SYPHILIS IN DIFFERENT COUNTRIES.

Rey, in *Annales de Dermatologie et de Syphiligraphie*, regards syphilis as at present an essentially universal disease. Two countries only in the known world are exempt, Iceland and the interior of Africa. In Iceland the physicians say that though frequently introduced it has never gained a foothold. That this is not due to climate is shown by the fact that syphilis is rife in such countries as Siberia and Greenland. According to Livingston the unmixed tribes of Central Africa do not have the disease, while it prevails among the mixed races. Those races, says Rey, suffer most were syphilis is introduced for the first time, the cases being of a graver character. The symptomatology of the disease is everywhere the same, except that of course the color of the skin has some influence on the character of the skin lesions. Climate is, on the whole, without essential influence. Authors are curiously at variance on this point, some considering hot climates favorable to the course of the disease, while others take the opposite view. Facts are wanting. It seems certain, however, that hot climates are bad for Europeans who may have contracted syphilis. In China the disease seems to run a peculiar and stubborn course. In Rey's opinion this probably is the result of improper treatment. In high latitudes syphilis runs an unfavorable course. Warm climates seem to favor the rapid effect of treatment.—*N. Y. Med. Times.*

